# Characterization of TauC3 antibody and demonstration of its potential to block tau propagation

**DOI:** 10.1371/journal.pone.0177914

**Published:** 2017-05-22

**Authors:** Samantha B. Nicholls, Sarah L. DeVos, Caitlin Commins, Chloe Nobuhara, Rachel E. Bennett, Diana L. Corjuc, Eduardo Maury, Bahareh Eftekharzadeh, Ololade Akingbade, Zhanyun Fan, Allyson D. Roe, Shuko Takeda, Susanne Wegmann, Bradley T. Hyman

**Affiliations:** Massachusetts General Hospital, Harvard Medical School, Department of Neurology, MassGeneral Institute for Neurodegenerative Disease, Charlestown, Massachusetts, United States of America; McGill University, CANADA

## Abstract

The spread of neurofibrillary tangle (NFT) pathology through the human brain is a hallmark of Alzheimer’s disease (AD), which is thought to be caused by the propagation of “seeding” competent soluble misfolded tau. “TauC3”, a C-terminally truncated form of tau that is generated by caspase-3 cleavage at D421, has previously been observed in NFTs and has been implicated in tau toxicity. Here we show that TauC3 is found in the seeding competent high molecular weight (HMW) protein fraction of human AD brain. Using a specific TauC3 antibody, we were able to substantially block the HMW tau seeding activity of human AD brain extracts in an in vitro tau seeding FRET assay. We propose that TauC3 could contribute to the templated tau misfolding that leads to NFT spread in AD brains.

## Introduction

In addition to its role as an axonal microtubule binding protein, tau is a major constituent of neurofibrillary tangles (NFTs) occurring in Alzheimer’s disease (AD) brains [1,2]. Recent data suggest that misfolded and modified tau can be taken up by neurons and act as “seed” for intracellular templated tau misfolding and the subsequent propagation of aggregating species [3–5]. Tau undergoes several post-translational modifications, and it is unclear which forms constitute the seed competent species. Soluble high molecular weight (HMW) tau isolated from human AD brain shows seeding bioactivity in in vitro assays [3,4] and contains various truncated and phosphorylated forms of tau.

Truncation of tau can be initiated by caspase-3, which cleaves tau at residue D421, producing the C-terminally truncated form of tau referred to as tauC3 (aa1-421) [6]. The activation of caspases occurs early in apoptosis and is also thought to be involved in the cascade of tau processing that eventually leads to NFT formation[6]. There are other putative caspase cleavage sites in the sequence of tau, but it has been confirmed that D421 is the predominant target for caspase-3[7]. In accord with previous observations of tau in the human CNS in AD [8], our initial characterization of tau present in the soluble fraction of extracts from AD brain, antibodies generated against the very C-terminal end of tau (residues 422–440) did not reliably recognize seeding competent HMW tau from these preparations derived from AD brain frontal cortex, suggesting that the C-terminus may be cleaved or inaccessible.

To further explore this observation, in this study we characterize the antibody, anti-TauC3, specific for TauC3 that has been described by Binder et. al [9]. We first show that anti-TauC3 recognizes tauC3 but not full-length tau, both in recombinant tau and AD brain extracts. We then evaluated the potential of tauC3 as a seeding competent species in human brains.

We find that anti-TauC3 is very specific for TauC3 compared to full-length recombinant Tau and confirm data demonstrating its usefulness as a reagent in detecting TauC3 in human brain tissue. Further, pre-treatment with anti-TauC3 largely blocks seeding from tau derived from AD brain. These studies lead us to believe that this C-terminally truncated form of tau, possibly a product of caspase cleavage, is likely a component of the bioactive HMW fraction responsible for tau seeding.

## Materials and methods

### Preparation of HMW AD extracts

Human AD brain tissue from Frontal Cortex (FC) was obtained from the Massachusetts Alzheimer’s Disease Research Center Brain Bank. The subject demographics are listed in Table 1. All of the subjects or their next of kin gave informed consent for their brain donation. The Massachusetts General Hospital Institutional Review Board approved the study protocol. The brain lysates for the study were prepared as previously described in Takeda et al. [4]. Briefly, brain cortical tissue was homogenized in 5x (w/v) ice-cold phosphate buffered saline (PBS) containing protease inhibitors (Cell Signaling 100X Protease Inhibitor Cocktail) and centrifuged at 4°C for 10 min at 10,000 x g. The supernatant was filtered through a 0.22 µm syringe filter (Corning), and separated on a Superdex 200 10x300 (GE Healthcare) size exclusion column. Fractions of 0.5 ml were collected starting at an elution volume of 6.5 ml. Fractions 2 and 3 (7.5 ml + 8.0 ml elution volume) containing the HMW tau were pooled and the tau concentration determined using a Total huTau ELISA (Invitrogen).

**Table 1:** Human case profiles.

Case Number	Age	Sex	PMI*	Neuropathological Diagnosis	Braak/Braak
1199	>90	F	12	Alzheimer's Disease	VI
1212	87	M	13	Alzheimer's Disease	VI
1222	90	F	5	Alzheimer's Disease	VI
1266	51	F	10	Alzheimer's Disease	VI
1877	>90	F	16	Alzheimer's Disease	VI
2026	>90	F	12	Alzheimer's Disease	VI

**Fig 1:**
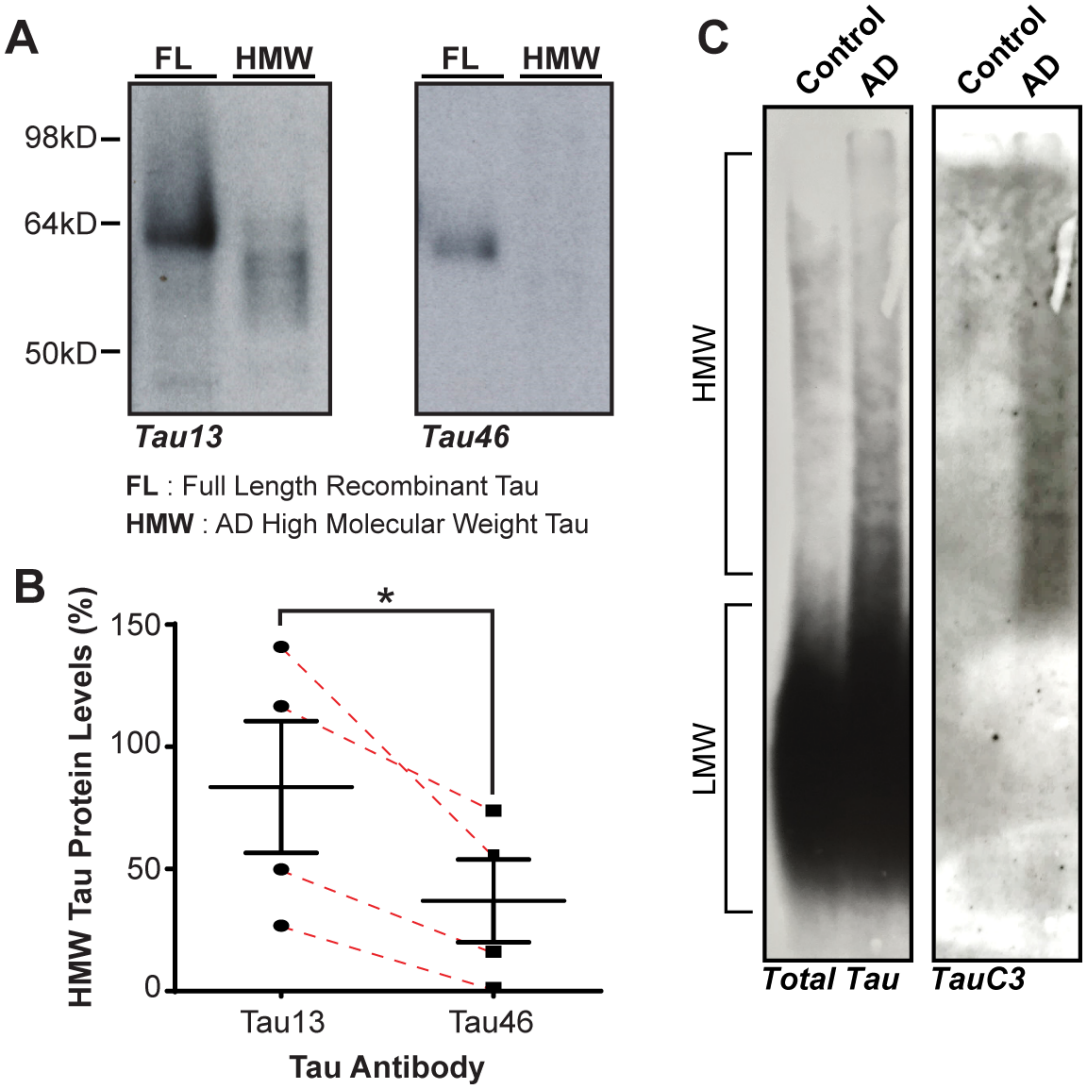
High molecular weight tau from Alzheimer’s disease brain is C-terminally truncated. **A**. Western blot probed with Tau46 (C-terminal Tau antibody) and Tau13 (N-terminal antibody) showing the HMW fraction isolated from human AD brain tissue does not contain the full-length Tau (441 aa). **B**. Quantification of Tau13 and Tau46 from AD HMW fractions (n = 4 AD cases), showing that the amount of HMW tau that contains the C-terminal Tau46 epitope is significantly lower than the Tau13 N-terminal epitope. Dashed red lines indicate the same human case. Absolute amounts are calculated by normalization against a recombinant human full length tau standard. **C**. SDD-AGE analysis of a Control and AD case for total tau and TauC3 show that only the AD case has TauC3 signal in the High Molecular Weight (HMW) fraction. Error bars represent Mean +/- SEM. Two-tailed student t-test. *p<0.05.

Human case lysate used throughout the study. Cases used in Fig 1: 1199, 1212, 1222, 1266, and 1877. Cases used in Fig 3: 1199, 1212, 1266, 2026.

*PMI: Post-mortem interval

### Expression and purification of recombinant tau species

Both recombinant forms of tau used (full-length tau (2N4R, aa1-441) and TauC3 (aa1-421); Table 1) were made by introducing stop point mutations to the pET29b full-length tau plasmid (Addgene plasmid # 16316) at the appropriate residues. The proteins were expressed in E.coli strain Bl21(DE3) as described in [10]. Briefly, protein expression was induced at OD 0.6 by adding 1 mM IPTG for four hours at 37°C. The bacteria were pelleted and resuspended in resuspension buffer [20 mM MES, 1 mM EGTA, 0.2 mM MgCl_2_, 5 mM DTT, pH 6.8] containing protease inhibitor cocktail (Roche) and phosphatase inhibitor cocktail (Sigma). Cells were disrupted by sonication and boiling for 20 min in high salt buffer [20mM MES, 1 mM EGTA, 0.2 mM MgCl_2_, 5 mM DTT, 500 mM NaCl, pH 6.8]. Cell debris and insoluble denatured proteins were pelleted by ultracentrifugation at 127,000 x g for 45 min at 4°C. This supernatant was used for SDS-PAGE and Western bot analysis. For Biacore experiments, the supernatant was dialyzed overnight at 4°C against [10 mM HEPES, 50mM NaCl, pH 7]. The dialysate was cleared by ultracentrifugation as above and loaded onto a cation exchange column (GE Healthcare). The recombinant proteins were eluted using a linear gradient over 6 column volumes to a final concentration of 60% 10 mM HEPES, 1 M NaCl. All tau protein concentrations were determined using a Total huTau ELISA (Invitrogen).

## Kinetic measurements by surface plasmon resonance

Measurements were taken using a Biacore T200 (GE Healthcare). Recombinant tauC3 or full-length tau were immobilized on a CM5 series S chip in running buffer (PBS, 0.01% Tween-20, pH 4.3) using an amine coupling kit (GE Healthcare) directed to a target RU of 150. Anti-TauC3 antibody (Intellect) [9] was flowed at concentrations ranging from 0.001 to 5 nM in running buffer. Each independent experiment included five different concentrations within this range and curves were compared to a reference cell that was mock immobilized (no protein was coupled, coupling reagents flowed as in experimental cells) as a baseline for non-specific binding. Experiments were performed on three separate days each with different batches of recombinant proteins.

### Immunodepletion

Tau immunodepletion from brain extracts was performed using Dynabeads Protein G Immunoprecipitation Kit (#10007D, Life Technologies) according to the manufacturer’s instructions with minor modifications. 0.75 mg of Dynabeads Protein G were incubated with 2 µg of each tau antibody or control IgG diluted in Binding & Washing buffer for 10 min with rotation at room temperature. Tau antibodies HT7 (mouse, Thermo Fisher Scientific #MN1000) and anti-TauC3 were compared against mouse IgG as a negative control. After washing with 100 µl of Binding and Washing buffer, the Dynabeads-antibody complex was incubated with 100 µl of human AD brain extracts (PBS-10,000 g, 2 µg/µl total protein) for 10 min with rotation at room temperature. Dynabeads-antibody-antigen complex was isolated using a magnetic holder and the supernatant was collected.

### Western blot analysis

Recombinant tau proteins and HMW protein in SEC fractions 2 and 3 from human brains were separated under reducing conditions in 4–12% Bis-Tris polyacrylamide gels (Life Technologies) using 1x MES SDS running buffer (Life Technologies). For anti-TauC3 antibody specificity, the Coomassie gel was stained with Simply Blue (Thermo Fisher). Proteins were transferred to polyvinylidene fluoride membrane (Thermo Fisher), and membranes were blocked for 1 hour at room temperature in blocking buffer (LI-COR), then incubated with anti-TauC3 antibody (1:1000, mouse, Intellect) and total tau antibody (1:2000, rabbit, Abcam ab39524) in LI-COR blocking buffer overnight at 4°C. After washing in Tris-buffered saline containing 0.25% Tween-20 (TBS-T) the membranes were incubated 1 h at room temperature in secondary anti-mouse IR800 and anti-rabbit IR680 (1:2000, LI-COR). After washing in TBS-T, the western blot membranes were imaged on an Odyssey Infrared Imaging instrument (LI-COR). For the high molecular weight protein fractions from human brain, protein was transferred onto PVDF membrane and probed with a C-terminal Tau antibody, Tau46 (Cell Signaling; epitope amino acids 404–441), or an N-terminal Tau antibody, Tau13 (Biolegend; epitope amino acids 2–18). Band quantification was performed using Image J densitometry analysis and the amount of tau per blot and antibody was normalized to a known amount of recombinant tau that was loaded on the same blot.

### Semi-denaturing detergent agarose gel electrophoresis (SDD-AGE)

For SDD-AGE, based on previous protocols [11,12], a 20 cm 1.5% agarose gel was poured with Buffer G (20mM Tris, 0.2M glycine) containing 0.02% sodium dodecyl sulfate (SDS). Samples were prepared with 50 µg total protein in 4X sample buffer (20% glycerol, 0.01% bromophenol blue, 0.08% SDS), incubated for 7 minutes at room temperature before being loaded onto the gel. The gel was run overnight at 30V on ice using Laemmli buffer (Buffer G + 0.1% SDS). Proteins were transferred to polyvinylidene difluoride (PVDF) membrane via capillary action overnight at 4°C. The resulting protein blots were blocked with 5% milk in TBS-T for 1 hour then probed first with mouse TauC3 antibody (1:1500, Intellect) overnight at 4°C and goat anti-mouse HRP (1:2000, Bio-Rad) for 1 hour at room temperature. After a brief incubation with chemiluminescent substrate (Pierce, ECL Western Blotting Substrate), blots were exposed to film (Kodak). Peroxidase was quenched using 1% sodium azide in TBS-T for one hour at room temperature. After confirming quenching via film exposure, blots were reprobed with a rabbit polyclonal tau antibody (1:4000, Abcam) overnight at 4°C, followed by goat anti-rabbit HRP and subsequent detection by film.

### TauFRET2 seeding assay

The TauFRET2 seeding assay is similar to a previously published HEK cell seeding assay [13]. In the TauFRET2 assay, the repeat domain of Tau (TauRD) [14] containing the pro-misfolding P301L mutation, is fused to CFP, followed by a self-cleaving 2A peptide [15] and a second TauRD fused to YFP (TauRD-CFP-2A-TauRD-YFP). This allows for the stoichometrical ~1:1expression of both TauRD-CFP and TauRD-YFP as individual proteins from a single plasmid (pcDNA3.1-TauFRET2). For the HEK293 seeding assay, HEK-293 cells were plated in DMEM containing 10% FBS in 96-well plates at 2.5x10^4^ cells/well. The following day, cells were transfected with 100 ng/well pcDNA3.1-TauFRET2 for 6 h using Lipofectamine 2000 (Invitrogen). AD brain extract (final concentration = 2 µg total protein/well diluted in OptiMEM) was pre-incubated with either 1% lipofectamine (positive aggregation control), anti-TauC3 antibody, anti-total human tau antibody HT7 (ThermoScientific), or PBS diluted in sterile OptiMEM at 37°C for 20 min. Transfected cells were carefully washed 2-times with PBS, the lysate-antibody solutions were added to the cells, and cells were incubated under culture conditions. After 60 h, cells were trypsinized, resuspended in cold DMEM with 10% FBS and pelleted at 1,000xg for 10 min in a plate centrifuge. Pellets were then resuspended for fixation in PBS containing 2% paraformaldehyde for 10 min at room temperature, pelleted as before, resuspended in cold PBS, and analyzed for Foerster resonance emission transfer (FRET) intensity (excitation with 405 nm laser, emission through 525/50 filter) using a Miltenyi VYB flow cytometer (Miltenyi). Using HEK293 non-transfected cells to draw the appropriate gate for CFP/YFP dual expressing cells, single CFP/YFP dual expressing HEK293 cells were analyzed for the presence of FRET. For the N2A seeding assay, N2A cells were plated in OptiMEM containing 5% fetal bovine serum (FBS) and penn/strep in 96-well plates at 3.5x10^4^ cells/well. The next day, cells were transfected with 150 ng/well pcDNA3.1-TauFRET2 for 6 h using Lipofectamine 2000 (Invitrogen). Transfected cells were washed 2-times with PBS, and IgG, anti-TauC3, or HT7 immunodepleted AD brain extract (final concentration = 2 µg total protein/well diluted in 50uL OptiMEM) was add to cells. Cells were incubated under culture conditions for 36 h, after which cells were trypsinized, resuspended in cold OptiMEM with 5% FBS and pelleted at 1,000xg for 10 min in a plate centrifuge. Cells were fixed and run on the flow cytometer the same was as the HEK293 cells. For FRET quantification, a bivariate plot of FRET vs. CFP was created to assess the percentage of FRET-positive cells, using TauFRET2 transfected cells that received no lysate as a marker of the FRET-negative population. The integrated FRET density (percentage of FRET-positive cells multiplied by the median fluorescence intensity of FRET-positive cells) was used for all quantitative analyses. Each experiment was performed using 20,000–50,000 cells per replicate and each condition was analyzed in at least triplicate. Data analyses were performed with MACSQuantify (Miltenyi). Representative images of tau aggregates in the HEK293 and N2A TauFRET2 transfected cells were taken using the EVOS fluorescent microscope (ThermoScientific) using the GFP channel.

### Data analysis and statistics

All statistical tests were performed using Graphpad Prism version 6 and all data are expressed as mean ±SEM. For the two-group comparison between Image J densitometry analysis of Tau13 and Tau46 HMW band quantification, a two-tailed Student’s t-test was used. Comparison between the relative tau protein levels and no lipofectamine treatment groups for seeding activity were performed by one-way analysis of variance (ANOVA) and post hoc Sidak multiple comparison test. The statistical test used is described in each figure legend. P values less than 0.05 were considered significant. Graphs are represented as mean +/- SEM.

## Results and discussion

### Detection of full-length tau and tauC3 in human AD brain extracts

Recently we found that HMW tau from human AD brain extracts can seed the aggregation of tau in cells in vitro [4]. To better understand which forms of tau are present in the HMW fraction, we probed western blots of this HMW fraction from human AD brain extract (Table 1) with antibodies recognizing the C-terminus (Tau46, epitope aa404-441) or the N-terminus (Tau13, aa2-18) of full-length tau. We found that the C-terminal antibody (Tau46) appeared to recognize less tau, and a different ensemble of tau proteins compared to the N-terminal antibody (Tau13) (Fig 1A), indicating that HMW AD tau contains truncated forms of tau, presumably C-terminally truncated. We also observed some variation in the tau composition between patients (Fig 1B), though there was consistently less Tau46 signal as compared to Tau13 signal in each patient. Next, we evaluated if C-terminally caspase-3 cleaved tau, TauC3, was present in HMW AD tau. TauC3 has previously been shown to cause mitochondrial stress when overexpressed in cells and neurons [6,14,16,17]. Using an antibody against the caspase-3 cleavage site at D421, anti-TauC3, we were able to detect TauC3 in the HMW protein fraction of AD brain on an aggregate preserving semi-denaturing detergent agarose gel electrophoresis (SDD-AGE) blot (Fig 1C).

### Anti-TauC3 antibody is specific for the caspase-3 cleaved form of tau

**Fig 2:**
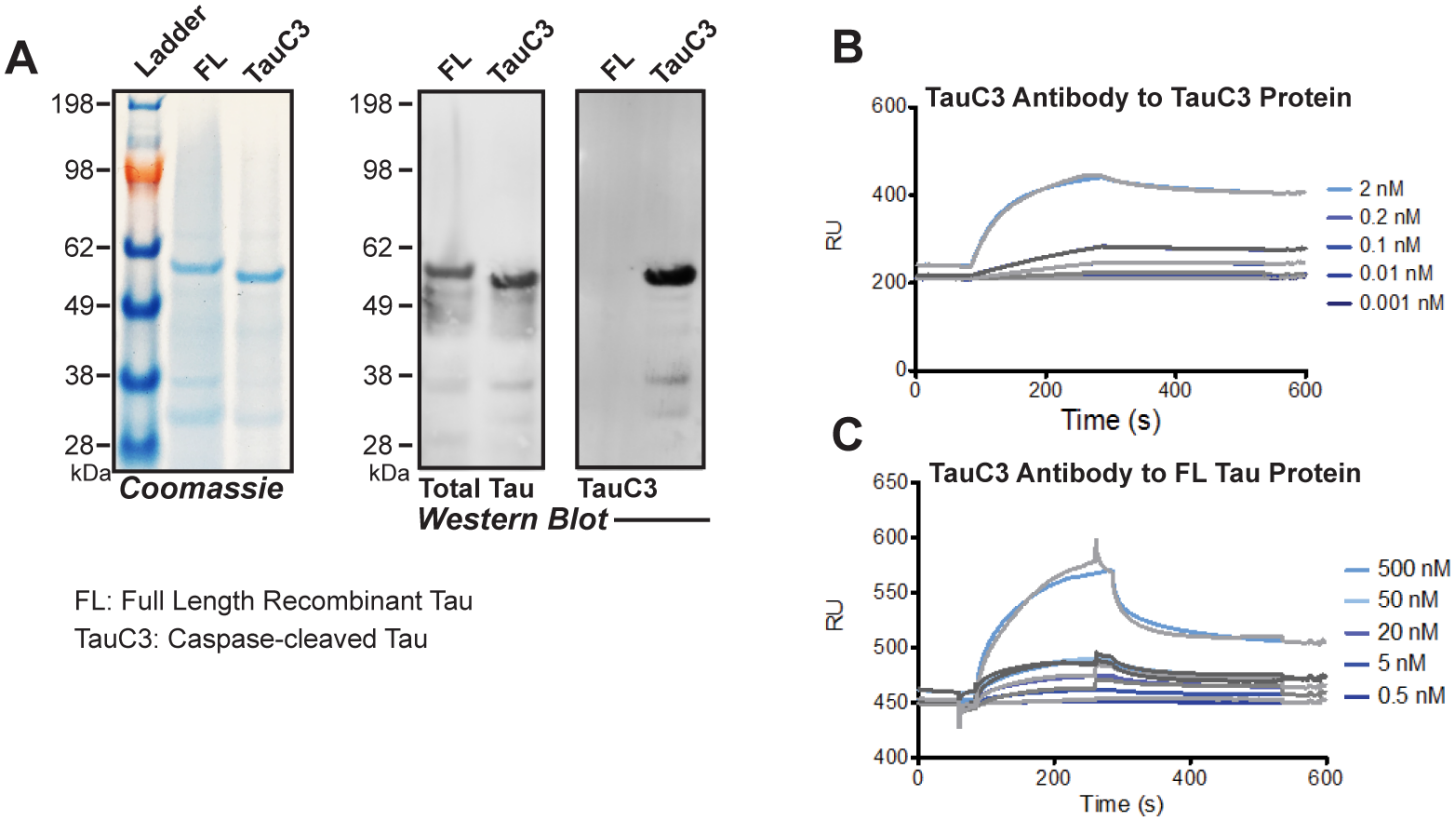
Anti-TauC3 antibody is highly specific for TauC3 protein. **A**. Coomassie stained SDS—PAGE (left) and western-blot (right) probing recombinant Tau species (the Tau repeat domain, the N-terminal domain, TauC3, and full-length tau) using Anti-TauC3 antibody. **B-C**. Representative SPR binding curves of the Anti- TauC3 antibody to recombinant TauC3 (B) and full-length 4RTau (C).

In order to evaluate the specificity of the anti-TauC3 antibody, a coomassie and western blot was run on recombinant full-length tau (FL) and TauC3 (aa1-421) (Table 2 and Fig 2A). antiTauC3 antibody successfully recognized only recombinant TauC3. Once we showed that that anti-TauC3 was specific for TauC3, the binding kinetics of anti-TauC3 to recombinant TauC3 were determined by surface plasmon resonance (SPR, Biacore) binding measurements. Because recombinant TauC3 tended to precipitate in the standard buffer conditions necessary for SPR experiments, we chose to immobilize recombinant TauC3 via amine coupling to a series S CM5 chip. When flowing anti-TauC3 antibody over the immobilized TauC3, an apparent kinetic binding (equilibrium dissociation constant KD) of 2 to 5 x10^-11^ M was determined (n = 3 independent experiments) (Fig 2B). For full-length tau, the anti-TauC3 antibody had an apparent KD of 4 x 10^−8^ M (Fig 2C). As the affinity is three orders of magnitude less than the KD for TauC3 protein, this confirms the specificity of the TauC3 antibody (Table 3).

**Table 2:** Forms of recombinant tau.

Tau Form	Number of Amino Acids	Molecular Weight
Full-length 4R Tau (2N4R)	441	45.7kDa
TauC3 (caspase cleaved Tau)	421	43.7kDa

Forms of recombinant tau protein that are used to assess the specificity of the Anti-TauC3 antibody.

**Table 3:** TauC3 antibody characteristics.

Recombinant Protein	ka(1/Ms)	kd(1/s)	KD (M)	Chi^2^ (RU^2^)
TauC3	11081785	0.000543	4.9 x 10^−11^	7.19
4RTau	49673	0.00209	4.21 x 10^−8^	19.58

Binding constants (ka (association rate), kd (dissociation rate), KD (equilibrium dissociation constant), Chi^2^ (measure of error in fit to the experimental curve)) of anti-TauC3 antibody as determined by Biacore kinetics. Measurements were taken using a Biacore T200 with the respective recombinant proteins immobilized on a CM5 series S chip as the ligand with varying concentrations of the Intellect anti-TauC3 antibody as the analyte.

### Anti-TauC3 antibody blocks seeding activity of AD lysate in a HEK cell tau aggregation assay

**Fig 3:**
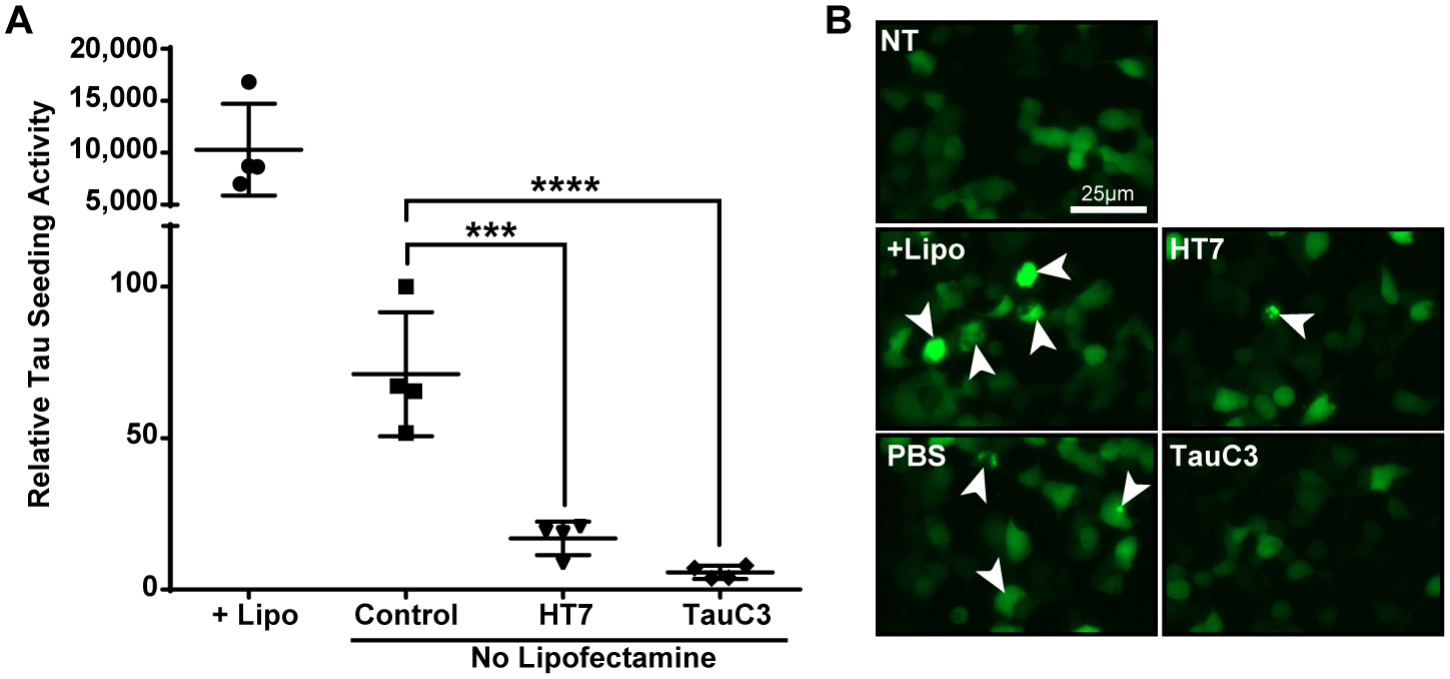
Anti-TauC3 antibody is efficient at blocking tau seeding activity in HEK293 cells. **(A)**. TauC3 positive AD lysate was pre-incubated with a PBS control, mid-domain tau (HT7), or TauC3 antibody and added to HEK293 cells that were transfected with the TauFRET2 construct. Cells were incubated with the lysate/antibody mixture for 60 hours and then analyzed on the flow cytometer. The relative tau seeding activity is the percent FRET positive cells multiplied by the median fluorescence intensity of the FRET-positive population, normalized to the PBS control mean. (B). Representative images of tau aggregates using the GFP channel. One-way ANOVA, Sidak post-hoc multiple comparisons. Error bars represent mean +/- SEM. ***p<0.001, ****p<0.0001.

To next assess whether the TauC3 that we detected in AD brain lysate is relevant for the cellular uptake and tau aggregation seeding of human AD brain extracts, we used a highly sensitive cell-based tau aggregation assay (TauFRET2) that we developed in analogy to an existing assay [13]. In this assay, HEK293 naïve cells are treated with TauFRET2 DNA (pcDNA3.1TauRD^P301L^–CFP/2A/TauRD^P301L^-YFP) so that the cells are expressing TauRD^P301L^-CFP and TauRD^P301L^-YFP equally throughout the cell. HEK293 cells expressing TauFRET2 are then treated with total human AD brain homogenate that contains the seed competent HMW tau. As a positive control to ensure that there were aggregates of tau that could induce seeding, the AD lysate plus 1% lipofectamine was added directly to the cells, triggering the aggregation of TauRD^P301L^-CFP/YFP in the cells, leading to FRET activity. We next pre-incubated AD brain lysate from four different AD brains with either PBS (negative control), HT7 (a mid-domain total-tau tau antibody), or TauC3 antibody and subsequently determined seeding activity measured as FRET intensity. We found that anti-TauC3 substantially and significantly reduced tau uptake/aggregation in HEK293 transfected cells by ~92% compared to control treatment, and appears to be at least as effective as the positive control HT7 total tau treated cells (Fig 3).

### TauC3 contributes to a significant amount of tau seeding activity in AD lysate using a neuronal aggregation assay

**Fig 4:**
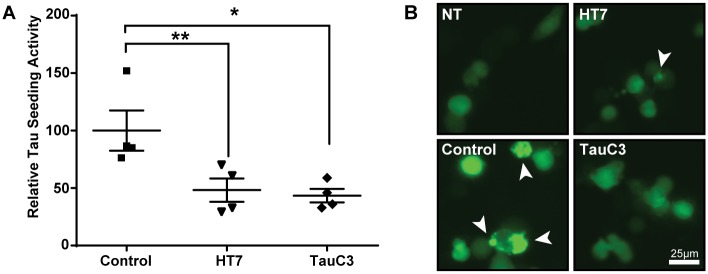
Anti-TauC3 antibody recognizes a significant portion of tau seeding activity from human AD lysate in a neuronal aggregation assay. (A). Immunodepleted fractions were added to TauFRET2 DNA transfected neuronal-like murine N2A cells for 36 hours and analyzed for FRET activity. The relative tau seeding activity is the percent FRET positive cells multiplied by the median fluorescence intensity of the FRET-positive population, normalized to the IgG immunodepleted sample mean. (B). Representative images of tau aggregates in N2A cells on GFP channel. One-way ANOVA, Sidak post-hoc multiple comparisons. Error bars represent mean +/- SEM. *p<0.05, **p<0.01, ***p<0.001, ****p<0.0001.

To evaluate whether the TauC3 that we detected in AD brain lysate is relevant in neuronal uptake and tau seeding activity, we first performed an immunodepletion on four human AD cases using a total tau antibody (HT7) and TauC3. Mouse IgG was used as a negative control. To test whether HT7 or TauC3 bound the bioactive species of tau, we utilized a similar assay as the TauFRET2 HEK293 cells, though instead of using HEK293 cells, we used the more disease relevant neuronal-like murine N2A cell line. N2A cells transfected with TauFRET2 were treated with immunodepleted samples—IgG (negative control), HT7 (total tau positive control), or TauC3 antibody—from each of the 4 AD cases, triggering the aggregation of TauRD^P301L^-CFP/YFP in the presence of seed competent tau aggregates, and leading to FRET signal in the cells. Human AD lysate that was immunodepleted of either total tau or TauC3 resulted in significantly less FRET positive tau aggregates in TauFRET2 expressing neuronal N2A cells (Fig 4). Together, the HEK293 and N2A seeding data imply that TauC3 is either directly present in the mix of tau species or closely interacts with the tau species in AD brains that are competent to induce tau seeding.

## Conclusions

Understanding the specific specie(s) responsible for tau seeding in the neurodegenerative cascade may be important in dissecting the molecular mechanisms that predispose to this process. Our current experiments explore the role of C-terminal truncated species in tau bioactivity of human AD brain extract. The data show that in AD lysate, the C-terminus recognized by the Tau46 antibody is not robustly apparent in the high molecular weight fraction (HMW) as assessed by Western blot of the HMW SEC fractions, indicating that the seed competent HMW fraction does not primarily include the full-length version of the protein (or that the epitope is inaccessible). These results are consistent with diminished C terminal peptides noted by mass spectroscopy in AD CSF [8]. C-terminal cleavage of tau by caspase-3 has been suggested to be an early event in tangle formation [6], and may be an early indicator of neurodegeneration. Recently Ozcelik et al. show that a TauC3 like protein Δtau (3R, aa 151–421), which is also truncated at D421, is toxic in mouse models at an early age [18], suggesting that truncated tau is a potential pathologically relevant species. As has been previously reported, we were able to detect TauC3 in tangles in AD tissue as well as that of AD-Downs patients (data not shown) [19], suggesting that TauC3 is present in the human AD brain.

The TauC3 antibody shows a very high binding specificity for the target caspase-cleaved Tau protein when tested against the recombinant TauC3 protein vs. the full-length protein in both western blotting and in SPR experiments. Despite the relative lack of abundance of tauC3 compared to total tau, the anti-TauC3 antibody was able to significantly block the seeding properties of AD lysate as assessed by the TauFRET2 seeding biosensor assay. We have previously shown [3,4] that depleting phosphorylated tau species similarly can provide inhibition of seeding seemingly out of proportion to their absolute amount in the mixture of tau present in the HMW fraction, and the current data with TauC3 provides an additional example of a dissociation between the apparent absolute amount of a post-translationally modified fraction of tau present in the HMW fraction, and the extent to which depleting that species blocks seeding.

It is thus probable that TauC3 is one of several species responsible for tau aggregation and seeding activity, or, at the very least, closely associated with the seed competent forms of tau. We suggest that further detailed characterization of the tau species present is warranted in determining specific therapeutic targets directed at the bioactive forms present. There are several reported cleavage sites upstream of the caspase cleavage site at D421 [20] which may determine a larger portion of the Tau species present in the HMW fraction, especially in later stage AD patients. The C-terminal region upstream of the caspase cleavage site (residues 243–406) has been shown to form protease resistant cores in tangles [21]. It is unknown if truncation occurs prior to oligomerization, or if a change in the relative accessibility of the C-terminus in an early misfolded tau molecule allows for truncation. Nonetheless, despite the large diversity of species present in the HMW fraction, the effectiveness at blocking seeding in the in vitro TauFRET2 assay—both in HEK293 cells and N2A cells—with a reagent specifically directed against the D421 neoepitope suggests that it is a component of the bioactive tau complex. These results may help guide further evaluation of the properties of propagating tau, and of the complex biology of different domains of the tau molecule in the context of seeding and aggregation.
